# Test–retest Reliability, Interrater Reliability, and Convergent Validity of the Targeted Box and Block Test in an Upper Extremity Prosthesis User Population

**DOI:** 10.1016/j.arrct.2025.100427

**Published:** 2025-01-13

**Authors:** Kimberly Kontson, Bin Wang, Nicole Leung, John M. Miguelez, Lauren Trent

**Affiliations:** a Office of Science and Engineering Laboratories, Center for Devices and Radiological Health, United States Food and Drug Administration, Silver Spring, MD; b Office of Clinical Evidence and Analysis, Center for Devices and Radiological Health, United States Food and Drug Administration, Silver Spring, MD; c Clinical Services, Advanced Arm Dynamics, Redondo Beach, CA

**Keywords:** Outcome measures, Prosthesis, Rehabilitation, Upper limb

## Abstract

**Objective:**

To provide evidence for test–retest reliability, interrater reliability, and convergent validity of the targeted Box and Block Test (tBBT) in the upper limb prosthesis user population.

**Design:**

An observational study was designed to assess various psychometric properties of the novel outcome measure. Participants completed the tBBT across 2 distinct testing sessions to assess test–retest reliability, which was quantified using the intraclass correlation coefficient (ICC) (3,k) and Pearson's correlation coefficient. Multiple raters scored the tBBT to assess interrater reliability, which was quantified using the ICC(2,k) and Pearson's correlation coefficient. Convergent validity was assessed by computing the Pearson's correlation coefficient between specific subtasks of the Capacity Assessment of Prosthesis Performance for the Upper Limb (CAPPFUL) and the tBBT.

**Setting:**

Clinic.

**Participants:**

A convenience sample of 20 transradial, unilateral upper limb prosthesis users.

**Interventions:**

Not applicable.

**Main Outcome Measures:**

tBBT, Box and Block Test (BBT), and CAPPFUL.

**Results:**

Interrater reliability for completion time and identification of unsuccessful transports were found to be excellent for the tBBT with ICC values of 0.97-0.99. Similarly, test–retest reliability was found to be good to excellent with ICC values >0.84. There were strong correlations between the scores obtained by different sessions and the scores given by different raters, with correlation coefficients exceeding 0.75. Moderate to strong correlations were found between the tBBT and BBT and subtasks of the CAPPFUL.

**Conclusions:**

The tBBT is a valid and reliable measure to assess the functional performance of individuals using an upper limb prosthetic device. This novel measure offers benefits of ease and speed of implementation; assessment of repetitive, ecologically representative movements; and quantification of performance using both speed and accuracy.

Improvement in upper limb function is an essential therapeutic goal for the upper limb prosthetic user population. There is an existing gap in population-specific outcome measures that can adequately quantify the complexities of the function of a prosthesis, yet are low in cognitive and physical demand. The recently developed targeted Box and Block Test (tBBT) is one example of an outcome measure that can address this gap.

The tBBT is a performance-based clinical outcome assessment tool that elicits ecologically representative actions, including movement initiation, grasp, transport, and controlled release of objects during an upper limb task.^1^ Two identical 0.25” thick inserts with target spaces arranged in a 3 × 3 pattern are placed in the tBBT box, with the 15.2” partition in the middle. Numbered blocks are placed in the target spaces on the side of the hand being tested. Participants are instructed to transport 1 block at a time, placing the blocks in the mirrored position on the opposite side of the partition. Participant performance in the tBBT is quantified by task completion time and the number of unsuccessful block transports, where an unsuccessful transport is any block that is not within the target space, leaning against the target grid, or any block that was dropped during the task. A previous study comparing the tBBT to the standard BBT and a modified version of the BBT suggested the tBBT elicits movements, which are more similar to those executed in everyday activities. Therefore, it may capture a more realistic assessment of how well an upper limb amputee operating a prosthetic limb, or a person with an upper limb disability, will function in the real world.^1^ Another benefit of the tBBT is its ease and speed of implementation, as well as its ability to assess the repetition of motion, all of which are essential for clinical use. The tBBT has been used to assess novel prosthesis control systems,^2^ evaluate robot performance during object manipulation tasks,^3^^,^^4^ and assess deep residual learning models in establishing sensor-based biometrics for user authentication.^5^

The collection of outcome measure data is an essential part of improving patient care, but it can place cognitive and physical burden on clinicians and patients.^6^ With low-burden measures such as the tBBT, the incorporation of outcome measure data collection into routine clinical care may be more sustainable. Although the tBBT shows promise as a research and clinical tool for upper limb assessment, this outcome measure has not been extensively validated in a clinical population of interest.

Reliability and validity are important measurement properties to assess. Reliability refers to the stability and consistency of scores from an assessment over time. In the context of performance-based outcome measures, validity refers to how accurately a test or measurement tool assesses what it is intended to measure.^7^ Strong validity means that the measure consistently and accurately reflects the specific outcome it aims to evaluate. Convergent validity refers to the degree to which scores from 2 theoretically related measures correlate with each other. It is a subtype of construct validity, which assesses how effectively an outcome measure assesses the concept that it is intended to measure.^7^ A preliminary investigation into the convergent validity of the tBBT in a small sample of upper limb prosthesis users was completed by the investigators.^8^ Results were encouraging, but consultation with patients and expert clinicians assisting in the development and evaluation of the measure led to design changes (see Methods) that required more complete validation before the measure could be confidently used in a clinical setting. Therefore, the goal of this study was to provide evidence for test–retest reliability, interrater reliability, and convergent validity of the revised tBBT in the upper limb prosthesis user population.

## Methods

### Participants

Participants were recruited through Arm Dynamics, a national network of clinics specializing in the care and prosthetic rehabilitation of people with upper limb loss or deficiency. Participants were eligible for the study if they were unilateral transradial or wrist disarticulation level amputees who used a myoelectric prosthesis with an electric hand. A total of 20 upper limb prosthesis users participated in the study. Basic demographic information as well as the tested terminal device, wrist components, time since amputation, and prosthesis usage for each participant is provided in table 1.

**Table 1:** Participant information

Participant ID	Age (y)	Age at Amputation (y)	Sex	Side of Amputation	Level of Amputation	Tested Terminal Device	Tested Wrist Component	With Prosthesis (y)	Average Daily Prosthesis Use (h/d)	Average Weekly Prosthesis Use (d/wk)
P001	26	23	Male	Right	TR	Bebionic hand	Electric wrist rotator	1.3	3	5
P002	46	44	Male	Right	TR	MyoHand variplus speed	Manually rotated wrist	0.8	6	7
P003	36	34	Male	Left	WD	TASKA hand	Manually rotated wrist	1.8	2	4
P004	42	Congenital	Male	Left	TR	Bebionic hand	Manually rotated wrist	7.0	6	7
P005	38	33	Male	Right	TR	TASKA hand	Electric wrist rotator	4.3	8	7
P006	52	44	Male	Right	TR	i-limb quantum	Electric wrist rotator	7.0	8	7
P007	39	34	Male	Right	TR	TASKA hand	Electric wrist rotator	4.2	<1	1
P008	44	31	Male	Left	TR	i-limb quantum	Electric wrist rotator	13.3	2	3
P009	43	32	Male	Right	TR	i-limb quantum	Manually rotated wrist	8.3	3	1
P010	47	42	Male	Left	TR	MyoHand variplus speed	Electric wrist rotator	5.3	3	2
P011	40	Congenital	Female	Left	TR	i-limb quantum	Electric wrist rotator	1.3	13	6
P012	78	69	Male	Left	TR	TASKA hand	Electric wrist rotator	2.8	8	7
P013	62	57	Male	Left	TR	Michelangelo hand	AXON wrist	4.6	1	7
P014	23	28	Male	Left	TR	TASKA hand	Flexion wrist	4.0	5	3
P015	63	56	Male	Right	TR	MyoHand variplus speed	Flexion wrist	5.6	5	7
P016	18	Congenital	Female	Right	TR	MyoHand variplus speed	Manually rotated wrist	3.0	7	6
P017	63	19	Male	Left	TR	Michelangelo hand	AXON wrist	5.8	10	3
P018	37	37	Female	Right	TR	Bebionic hand	Quick disconnect wrist	2.8	1	4
P019	27	Congenital	Female	Right	TR	Bebionic hand	Manually rotated wrist	1.9	6	7
P020	55	Congenital	Female	Left	TR	TASKA hand	Manually rotated wrist	2.0	6	7

TR = transradial, WD = wrist disarticulation.

All participants provided written informed consent before participation under the approved FDA IRB study protocol (IRB#16-071R). The FDA served as the IRB of record under the executed IRB Authorization Agreement with Arm Dynamics.

### Tasks

#### Targeted box and block test

**Fig 1:**
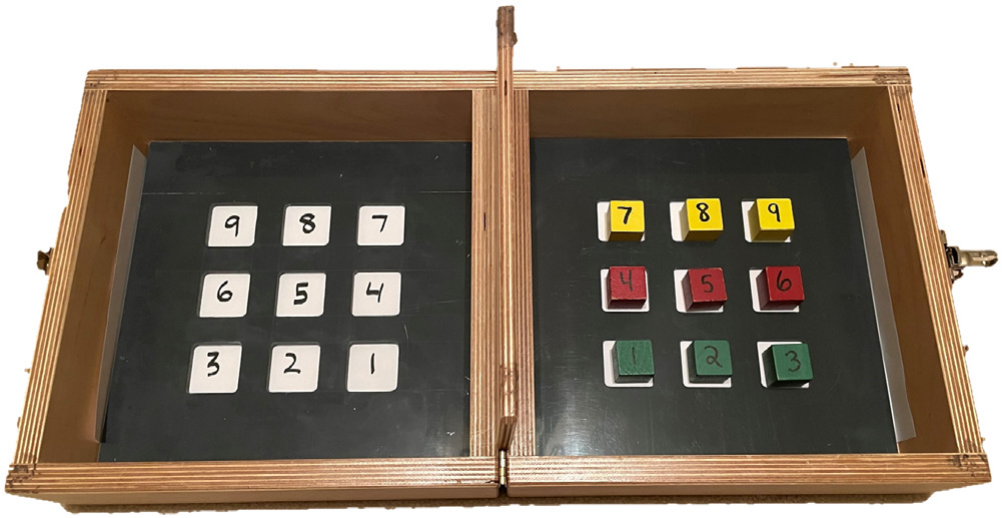
Setup of targeted Box and Block Test (tBBT).

A box with similar dimensions as the standard BBT was used for this assessment (53.7 × 17.2 cm box with a wall height of 8.5 cm and central partition height of 15.2 cm). The blocks were arranged in a 3 × 3 array on the side of the testing hand, using an insert with 9 numbered squares as a guide (fig 1). An identical insert was placed on the opposite side.

Participants were instructed to transport 1 block at a time, starting with block 1, closest to the partition in the row closest to the participant, placing the blocks in the mirrored position on the opposite side of the partition into target locations defined by an insert identical to the one on the side of the testing hand. The participant's score was recorded as the time taken to transport all 9 blocks. A stopwatch or timer was used to measure the completion time. According to publicly available tBBT instructions manual and video,^9^^,^^10^ the timer was started when an administrator says “Go” and stopped when the participant released the 9th block on the nontesting side of the partition. Administrators also kept track of those blocks that were considered unsuccessful transports (ie, blocks that were not fully placed into the target space and/or dropped blocks).

The development of the tBBT was an iterative process that involved consultation with expert researchers, prosthesis users, and clinicians specializing in the care and rehabilitation of upper limb prosthesis users. The preliminary designs were tested in the intended user population.^8^ Consultation with clinicians and patients after these preliminary tests resulted in a few key design changes to the tBBT. Specifically, the 4 × 4 block pattern was reduced to a 3 × 3 block pattern to give prosthetic users more space to manipulate blocks and decrease physical and cognitive demands on the patient. The numbering of blocks and target spaces was also introduced to reduce mental demand during the task so that the assessment would focus on measuring functional performance. These modifications allow the tBBT to more adequately assess functional movement while still maintaining the lower cognitive, temporal, and physical demands of the test. These modifications make the test better suited for this user population because it can easily be administered during therapeutic treatment as a complement to other patient performance and patient report measures.^11^

#### Box and block test

In the BBT, one hundred and fifty 2.5 cm^3^ wooden blocks are placed on the side of the partition with the testing hand in randomized orientations. A subject's score is equal to the number of these blocks transported over a 15.2 cm tall partition in 1 minute.^12^ The subject can select blocks in any order to transport over the partition as quickly as possible, with the only requirement being that the subject's fingertips cross the vertical plane of the partition. The BBT does not require controlled placement of the blocks on the opposite side of the partition. This measure has been extensively validated in numerous clinical populations with upper limb impairment.^13^ Additionally, it was determined that the BBT was responsive to change during upper limb prosthesis user training.^14^

The Capacity Assessment of Prosthetic Performance for the Upper Limb (CAPPFUL) is a performance-based outcome measure developed to assess the function of an upper limb prosthesis compared to that of a sound upper limb.^15^^,^^16^ It consists of 11 functional tasks for the prosthesis user to perform which are scored in several domains, including control skill, component utilization, and compensatory movement. The assessment also records the time required to perform each task, not to exceed 90 seconds. For the purposes of assessing convergent validity, the completion time for the tBBT was compared to the completion time for 4 CAPPFUL tasks that required targeted grasping, controlled transport, and release of smaller objects: Task 1 (weights in crate), Task 4 (pick up dice), Task 6 (lift plate), and Task 10 (coin in slot). The previous psychometric evaluation of the CAPPFUL in the upper limb prosthesis user population indicated that the measure had good interrater reliability, internal consistency, known-group validity, and convergent and discriminant validity.^15^

### Experimental protocol

A total of 10 raters across 5 study sites were trained on the administration of the tBBT through publicly available instructional materials^10^ and videos.^9^ To assess convergent validity, participants were asked to perform 3 trials of the standard BBT in a seated position, 3 trials of the tBBT in a standing position, 3 trials of the tBBT in a seated position, and 1 trial of the CAPPFUL in the first session of the study. The standard BBT assessment was scored using the recommended conventions in the cited literature above. The order in which all tasks were performed during the first session was counter-balanced.

To assess interrater reliability, 2 administrators scored all trials of the tBBT during the first session. If 2 administrators were not present during the time of testing, a video recording of the test box was obtained as the participant performed all trials of the tBBT. Special instructions were provided to all administrators to ensure the video was set up such that all blocks being transported could be viewed.

To assess test–retest reliability, participants were asked to return later during the same day or within 1 week of the first testing session. Because many participants travel long distances for their clinical appointments, requesting their return several days later to perform a single test was not a viable option. For those testing sessions done in the same day, the time between tBBT testing was maximized by providing participants time to rest and reset and/or having them complete other aspects of their clinical visit (eg, complete other outcome measures and discuss issues with their clinician). There was a strict minimum of 1 hour between testing sessions during which the patient rested to reduce fatigue, and other measures were administered to decrease the learning effect. All efforts were made to increase the time between testing sessions as much as possible.

### Statistical analysis

Outliers were defined as trial scores >q3+1.5 × (q3-q1), where q1 and q3 are the 25th and 75th percentiles of the outcome measure scores. Based on this definition, one participant (P002) was excluded from the final analysis because all their scores were identified as outliers as well as 2 individual trials from P006 to P008. Additionally, the video recording for the third tBBT standing trial for P005 was lost, so these data are categorized as NaN in the final analysis dataset.

The mean of the 3 trials for tBBT stand, tBBT sit, and BBT was computed for each participant.

The test–retest reliability was assessed using the ICC(3,k), which employs a 2-way mixed effects model assessing absolute agreement using the mean of multiple measurements.^17^ Pearson's correlation coefficient was also computed between the mean of the tBBT trials across sessions.

Interrater reliability was assessed using the ICC(2,k), which employs a 2-way random effects model for absolute agreement from multiple measurements. As described in Koo et al^17^ the multiple rater/measurement model was selected, given that the tBBT score in actual implementation will be the average of 3 trials from a single rater. Pearson's correlation coefficient was also computed between the scores for each rater. The interrater reliability was determined for completion time and identification of unsuccessful transports across raters.

Convergent validity was assessed by computing the Pearson's correlation coefficient between the mean of the tBBT trials and the mean of the BBT trials as well as the completion times for Tasks 1, 4, and 6 of the CAPPFUL. Because of the complexity of CAPPFUL Task 10 (coin in slot), only 1 participant successfully completed the task. Therefore, CAPPFUL Task 10 was removed from the convergent validity analysis. The subset of tasks from CAPPFUL was selected to best match the tBBT construct, ie, the manipulation of small objects. It was hypothesized that performance would correlate moderately on these tasks given the differences in the objects being manipulated and the location around the body in which the object manipulation occurred. Correlations between the BBT and tBBT were expected to be stronger (albeit negative because of the scoring approaches) given the similarities in the tasks.

An additional analysis was performed similarly to ICC(3,1) to evaluate the consistency of the tBBT scores across different raters and different sessions by computing ICC values without averaging trials, thereby offering a more granular understanding of variability and reliability. This ICC employed a 2-way mixed effects model where study participants were treated as random factors to account for individual differences. The coefficient of variation was also calculated to assess the consistency of scores. The normality of the tBBT scores in each of the trials was assessed using the quartile–quartile-plot and the Shapiro–Wilk test. Further details on these analyses as well as the results are provided in Supplemental Appendix S1 (available online only at http://www.archives-pmr.org).

For all statistical tests, α=0.05. ICC values were interpreted as follows: ICC<0.5 indicates poor reliability, 0.5≤ICC<0.75 indicates moderate reliability, 0.75≤ICC<0.9 indicates good reliability, and ICC≥0.9 indicates excellent reliability.^17^ Pearson's correlation coefficients were interpreted as follows: *R*<0.4 indicates weak correlation, 0.4≤*R*<0.7 indicates moderate correlation, and *R*≥0.7 indicates strong correlation.^18^

## Results

**Fig 2:**
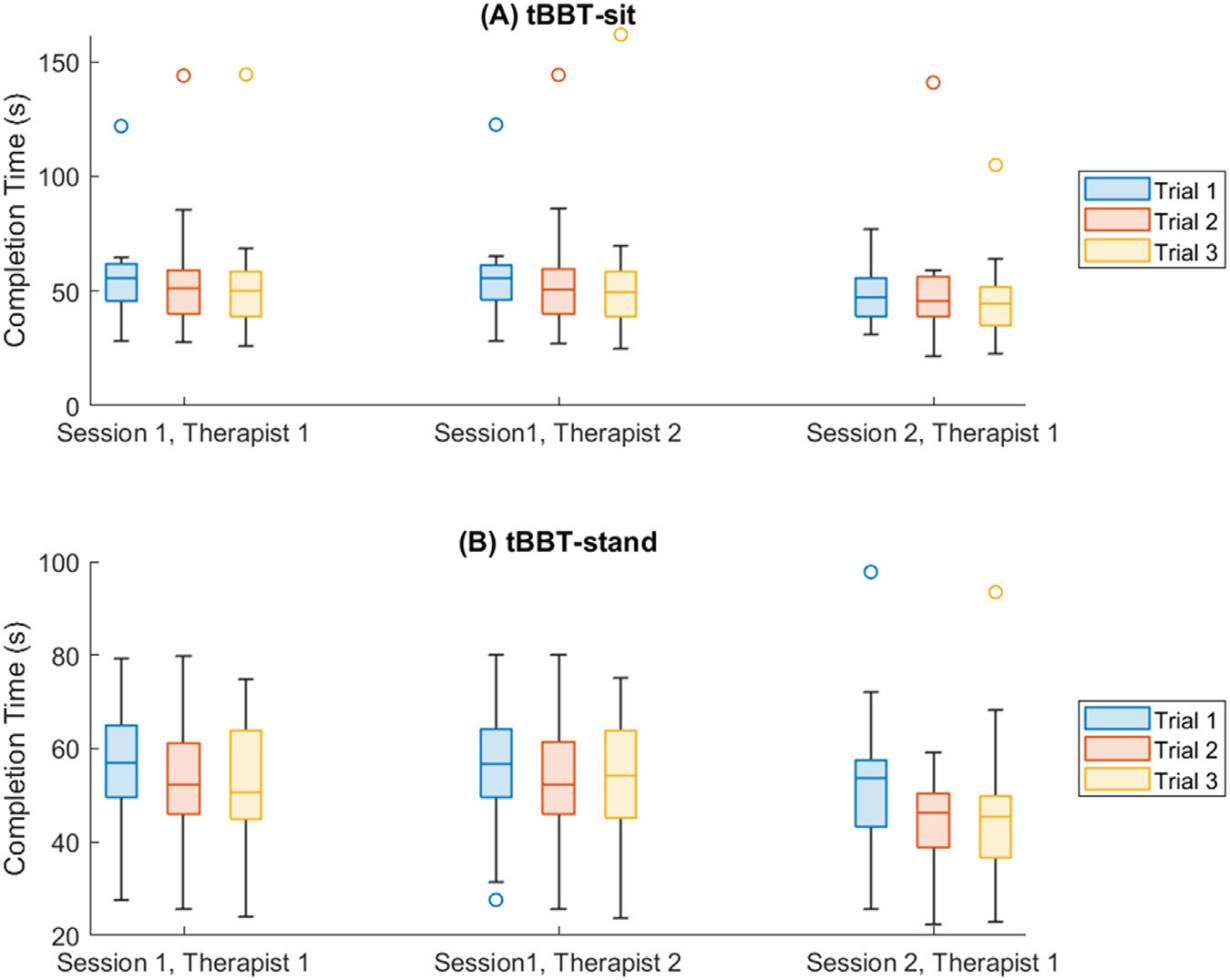
Boxplots showing distributions of completion time for each trial completed by participants for the (a) targeted Box and Block Test (tBBT) sit and (b) tBBT stand. Color circles indicate outliers that were removed from the final analysis.

Figure 2 shows the completion times for all 3 trials computed for the tBBT in the seated and standing positions across 2 sessions and 2 different raters.

Table 2 shows the results for test–retest reliability and interrater reliability for the tBBT in a standing and seated position. Interrater reliability for completion time and identification of unsuccessful transports were found to be excellent for both the seated and standing administration of the tBBT with ICC values of 0.97-0.99. Similarly, test–retest reliability was found to be good to excellent with ICC values of 0.84 and 0.90 for tBBT stand and tBBT sit, respectively. Similar results are seen with the 2-way mixed effects model for interrater reliability, with ICC values of 0.87 and 0.86 for tBBT sit and tBBT stand, respectively (supplemental table S1, available online only at http://www.archives-pmr.org/). Test–retest reliability was found to be moderate using the ICC mixed effects model (see supplemental table S1).

**Table 2:** Test–retest reliability and interrater reliability results for the seated and standing administrations of the tBBT

Task	Test–Retest Reliability				Interrater Reliability Completion Time				Interrater Reliability Unsuccessful Transports			
	*R*	*P*	ICC(3,k)	*P* (CI)	*R*	*P*	ICC(2,k)	*P* (CI)	*R*	*P*	ICC(2,k)	*P* (CI)
tBBT sit	0.82	<.001	0.90	<.001 (0.74-0.96)	0.99	<.001	0.99	<.001 (0.99-0.99)	0.97	<.001	0.98	<.001 (0.98-0.99)
tBBT stand	0.75	<.001	0.84	<.001 (0.58-0.94)	0.99	<.001	0.99	<.001 (0.99-0.99)	0.99	<.001	0.99	<.001 (0.98-0.99)

For both the standing and seated administration of the tBBT, there were also strong correlations between scores across sessions and scores determined by separate raters, with *R* values >0.75.

The evidence of convergent validity was also strong when the tBBT was compared to the BBT. Table 3 shows the Pearson's correlation coefficient for both the seated and standing positions. Given the strong interrater reliability, only the completion times recorded by therapist 1 were used to compare the BBT completion time scores. A moderate to strong negative correlation was found. Negative correlations were expected because higher completion times and lower block transport were both indicative of lesser performance. Convergent validity between the tBBT and CAPPFUL Task 1 (weights in crate) was not supported because the correlations were weak. However, there were moderate correlations between the tBBT and CAPPFUL Task 4 (pick up dice) and CAPPFUL Task 6 (lift plate).

**Table 3:** Convergent validity

Task	BBT		CAPPFUL Task 1		CAPPFUL Task 4		CAPPFUL Task 6	
	*R*	P	*R*	*P*	*R*	*P*	*R*	*P*
tBBT sit	−0.63	.004	0.28	.25	0.46	.18	0.65	.04
tBBT stand	−0.85	<.001	0.25	.31	0.62	.06	0.65	.04

## Discussion

Convergent validity was supported for the tBBT using 2 validated measures for the upper limb prosthesis user population. The subset of tasks from CAPPFUL was selected to best match the tBBT construct, ie, the manipulation of small objects. We hypothesized that performance would correlate with these tasks. Although moderate correlations between the completion times of the tBBT sit and stand were found between the CAPPFUL tasks of lifting a plate and picking up dice, low correlations were found between tBBT and the weights in the crate task (Task 1). The CAPPFUL Task 1 requires users to pick up weights on a table and place them into a crate on the floor, which expands the functional space within which the prosthesis user is required to operate their device.^15^^,^^16^ This contrasts with the functional space directly in front of the body within which the tBBT requires object manipulation. Although the CAPPFUL Task 1 provides an excellent example of the types of assessments needed to better characterize function of prosthesis users,^19^ the additional movements of rotating the body and bending down to place the weight in the crate on the floor result in the assessment of a different construct.

The tBBT provides several advantages over the traditional BBT. Specifically, it allows for quantitative study of initiation, grasping, and targeted transport of objects, and also of controlled object release, all of which are deemed important in object manipulation assessments.^20^ This enables test administrators to assess the user's prosthesis control ability more aptly and completely. The administrator can observe any maladaptive movement caused by the specific placement of blocks in space, as well as clear delineation of control and consistency in grasp and release. The simplicity of the measure is also a great benefit to clinicians and patients alike. The burden is low on both the test administrator for test setup and assessment and the user for test instructions and comprehension of the outcome score. With the current trend toward health care transparency and the desire for patients to be more involved in and informed of decisions relating to their health,^21^ the use of outcome measures that are easily understood and show clear progress or regression are key.

In addition to measuring the amount of time to complete a task, which is similar to how most upper limb outcome measures assess performance,^19^^,^^22^ the tBBT also includes a quantification of unsuccessful transports. The interrater reliability on unsuccessful transports was excellent, demonstrating that the written instructional materials^10^ and video instructions^9^ available to test administrators are adequate.

Although this outcome measure was developed for use by the upper limb prosthetic user population, it has the potential to be used and validated for other populations as well. This practice of applying an outcome measure to a population other than the one in which the measure was developed and validated is common in therapeutic populations that often have the same obstacles to overcome.^23^ This outcome measure is generalizable to other diagnoses that affect upper limb function such as TBI, stroke, and hand or arm injuries.

The evidence generated through this work supports the validity of the tBBT in assessing performance during the use of myoelectric prosthetic devices. Although test–retest reliability, interrater reliability, and convergent validity represent important measurement properties of a performance-based outcome measure, additional measurement properties should be explored to assess the ability of the measure to distinguish between different groups and assess change in performance over time. Responsiveness, in particular, is a very important measurement property, instilling confidence in a measure's ability to assess the effectiveness of a treatment and training regimen, as well as track a patient's overall progress.^24^ The evidence of responsiveness is lacking, in general, for outcome measures for upper limb prosthesis users,^14^ so future work should focus on this assessment.

### Study limitations

The current study only included myoelectric prosthesis users, which could limit the generalizability of the results. Additional testing in participants using other types of prosthetic devices may be necessary. Upper limb prosthesis users are difficult to recruit for such studies because of the paucity of the population. Although a larger sample size would have been preferred, statistically significant results were found for all psychometric properties assessed in this protocol. Additional research may also be needed to determine whether any correlation between prosthesis control skill and tBBT scores exists. Lastly, the use of a test–retest protocol completed in the same day may not generate the same degree of consistency for data separated by multiple days.

## Conclusions

The study has provided evidence supporting the validity and reliability of the tBBT as a measure for assessing the function and performance of individuals using an upper limb prosthetic device. This novel measure offers benefits of ease and speed of implementation; assessment of repetitive, ecologically representative movements; and quantification of performance using both speed and accuracy. The results from this study show the effectiveness of publicly available instructional materials and videos in conveying the necessary information to adequately administer and score the measure. The tBBT is a useful tool to incorporate into clinical care and research and development efforts for upper limb prosthesis users and would benefit from validation in additional clinical populations.

## Disclosure

None.

## Appendix Supplementary materials

Image, application 1

## Footnotes

Supplementary material associated with this article can be found, in the online version, at doi:10.1016/j.arrct.2025.100427.
