# Supplementary material for: Test–retest Reliability, Interrater Reliability, and Convergent Validity of the Targeted Box and Block Test in an Upper Extremity Prosthesis User Population
Source: Arch Rehabil Res Clin Transl. 2025 Jan 13;7(3):100427. doi: 10.1016/j.arrct.2025.100427 (PMC12447201; doi:10.1016/j.arrct.2025.100427)
Supplement: Supplementary file 1 [file mmc1.docx]

Appendix A – Additional statistical analyses

Contents

[ICC for mixed effects models 1](#_Toc177998270)

[Normality check 2](#_Toc177998271)

[Coefficient of Variation (CV) 4](#_Toc177998272)

# ICC for mixed effects models

Additional analyses were done to assess test-retest reliability and inter-rater reliability through inclusion of all three trials from each participant rather than averaging the three trials, as done in clinical practice. Inter-rater ICC coefficients were computed using mixed effects models by fitting the tBBT scores (sit or stand) from the three trials between the two session (for test-retest reliability) and between the two therapists (for inter-rater reliability), respectively, with subjects as a random factor [1-3]. The resulting ICC and confidence interval are reported in Table A1. The confidence level of the confidence interval is 95%.

References:

1. *Daniel L., Mattan S.B., Indrajeet P., Philip W. and Dominique M. (2021). performance: An R Package for Assessment, Comparison and Testing of Statistical Models. Journal of Open Source Software, 6(60),3139.*
2. *Hox, J. J. (2010). Multilevel analysis: techniques and applications (2nd ed). New York: Routledge.*
3. *Nakagawa, S., Johnson, P. C. D., and Schielzeth, H. (2017). The coefficient of determination R2 and intra-class correlation coefficient from generalized linear mixed-effects models revisited and expanded. Journal of The Royal Society Interface, 14(134), 20170213.*

Table A1: ICC for mixed effects models, where CI = 95% confidence interval

|  | Test-retest reliability | | Inter-rater reliability | |
| --- | --- | --- | --- | --- |
|  | ICC | [CI] | ICC | [CI] |
| tBBT sit | 0.783 | [0.751, 0.884] | 0.873 | [0.845, 0.943] |
| tBBT stand | 0.670 | [0.666, 0.823] | 0.857 | [0.841, 0.916] |

# Normality check

Normality of the tBBT scores in each of the nine trials were assessed using the quartile-quartile (QQ)-plot and the Shapiro-Wilk test. The QQ-plots can be used to visually check the normality and are shown in figures A1 and A2 below for tBBT-sit and tBBT-stand, respectively.


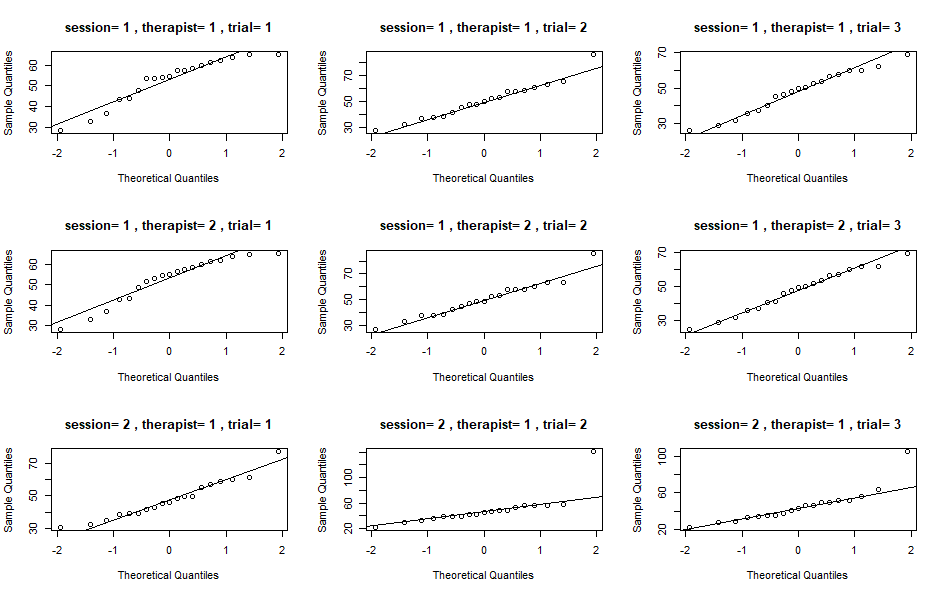


Figure A1: QQ-plot for tBBT-sit.


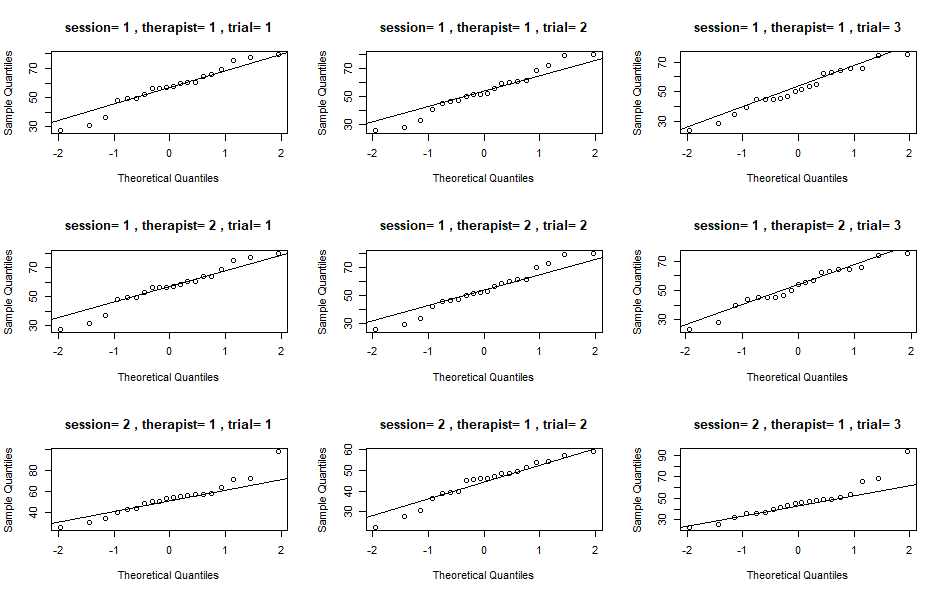


Figure A2: QQ-plot for tBBT-stand.

The p-values are also provided below for the normality tests using the Shapiro-Wilk test (Table A2). Column 4 shows the p-values with all scores, while the last column shows the p-values after removing all scores for “S002” and the outliers of “S006” and “S008” in two trials. Normality criteria are met for all trials except trial 1 for Session1/Therapist1.

Table A2: P-values for the Shapiro-Wilk normality test

| tBBT-sit | | | | |
| --- | --- | --- | --- | --- |
| Session | Therapist | Trial | p-value  (with outliers) | p-value  (w/o outliers) |
| 1 | 1 | 1 | <0.001 | 0.041 |
| 1 | 1 | 2 | <0.001 | 0.739 |
| 1 | 1 | 3 | <0.001 | 0.772 |
| 1 | 2 | 1 | <0.001 | 0.073 |
| 1 | 2 | 2 | <0.001 | 0.497 |
| 1 | 2 | 3 | <0.001 | 0.909 |
| 2 | 1 | 1 | 0.581 | 0.500 |
| 2 | 1 | 2 | <0.001 | 0.559 |
| 2 | 1 | 3 | <0.001 | 0.985 |
| tBBT-stand | | | | |
| Session | Therapist | Trial | p-value  (with outliers) | p-value  (w/o outliers) |
| 1 | 1 | 1 | 0.474 | 0.474 |
| 1 | 1 | 2 | 0.757 | 0.757 |
| 1 | 1 | 3 | 0.702 | 0.702 |
| 1 | 2 | 1 | 0.500 | 0.500 |
| 1 | 2 | 2 | 0.797 | 0.797 |
| 1 | 2 | 3 | 0.662 | 0.662 |
| 2 | 1 | 1 | 0.241 | 0.719 |
| 2 | 1 | 2 | 0.381 | 0.381 |
| 2 | 1 | 3 | 0.032 | 0.685 |

# Coefficient of Variation (CV)

The CV (=SD/mean) was computed for each subject in all nine trials. Histograms of the CV were plotted to visualize the distribution of CVs of sit and stand scores (Figure A3). A summary of the histograms is provided in Table A3. Based on these results, the variability of tBBT scores for both the sit and stand administrations is low.


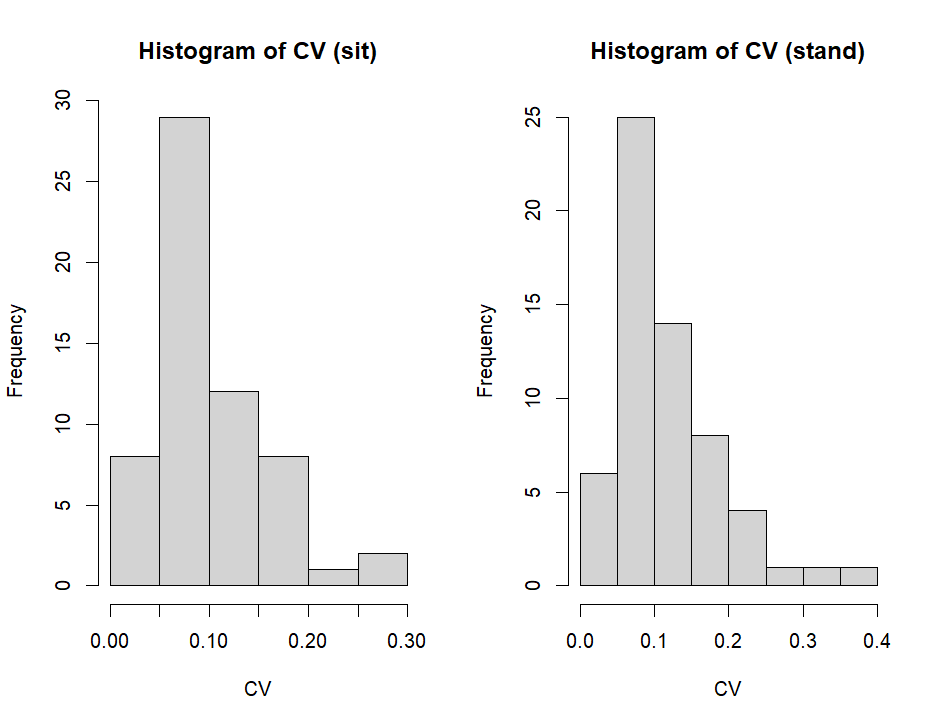


Figure A3: Histograms of CV for tBBT-sit (left) and tBBT-stand (right)

Table A3: Summary of histograms for CV

|  | **Min** | **1^st^ quartile** | **Median** | **Mean** | **3^rd^ quartile** | **Max** |
| --- | --- | --- | --- | --- | --- | --- |
| **tBBT-sit** | 0.0179 | 0.0631 | 0.0781 | 0.0987 | 0.1325 | 0.2980 |
| **tBBT-stand** | 0.0275 | 0.0639 | 0.0952 | 0.1134 | 0.1459 | 0.3559 |
